# Genetically engineered membrane-based nanoengagers for immunotherapy of pancreatic cancer

**DOI:** 10.1186/s12951-024-02369-9

**Published:** 2024-03-11

**Authors:** Haoqi Zhang, Yuanke Li, Helong Kang, Jingping Lan, Lin Hou, Zhengbang Chen, Fan Li, Yanqin Liu, Jiliang Zhao, Na Li, Yajuan Wan, Yiping Zhu, Zhen Zhao, Hongkai Zhang, Jie Zhuang, Xinglu Huang

**Affiliations:** 1 State Key Laboratory of Medicinal Chemical Biology, Key Laboratory of Bioactive Materials for the Ministry of Education, College of Life Sciences, and Frontiers Science Center for Cell Responses, Nankai University, Tianjin, 300071 China; 2 School of Medicine, Nankai University, Tianjin, 300071 China; 3 Key Laboratory of Molecular Biophysics of Hebei Province, Institute of Biophysics, School of Health Sciences and Biomedical Engineering, Hebei University of Technology, Tianjin, 300401 China

**Keywords:** Nanoengagers, Macrophages, Cell membrane, Pancreatic cancer

## Abstract

Modulating macrophages presents a promising avenue in tumor immunotherapy. However, tumor cells have evolved mechanisms to evade macrophage activation and phagocytosis. Herein, we introduced a bispecific antibody-based nanoengager to facilitate the recognition and phagocytosis of tumor cells by macrophages. Specifically, we genetically engineered two single chain variable fragments (scFv) onto cell membrane: anti-CD40 scFv for engaging with macrophages and anti-Claudin18.2 (CLDN18.2) scFv for interacting with tumor cells. These nanoengagers were further constructed by coating scFv-anchored membrane into PLGA nanoparticle core. Our developed nanoengagers significantly boosted immune responses, including increased recognition and phagocytosis of tumor cells by macrophages, enhanced activation and antigen presentation, and elevated cytotoxic T lymphocyte activity. These combined benefits resulted in enhancing antitumor efficacy against highly aggressive “cold” pancreatic cancer. Overall, this study offers a versatile nanoengager design for immunotherapy, achieved through genetically engineering to incorporate antibody-anchored membrane.

**Supplementary Information:**

The online version contains supplementary material available at 10.1186/s12951-024-02369-9.

## Introduction

Pancreatic cancer remains a lethal malignancy with an extremely low 5-year overall survival rate and a high recurrence rate [1–3]. Although surgical intervention combined with chemotherapy, such as gemcitabine and FOLFIRINOX, has shown incremental improvements in survival for pancreatic cancer [4], the majority of patients are diagnosed at an advanced stage, rendering them ineligible for surgery [5–7]. In recent years, immunotherapeutic strategies, such as checkpoint inhibitors and therapeutic cancer vaccines, have exhibited remarkable promises in treating various solid tumors [8, 9]. However, their application in “cold” pancreatic cancer has been challenging due to its immunosuppressive tumor microenvironment and low mutational burden, resulting in poor immune cell infiltration and limited tumor-specific antigen generation [10–12]. Thus, it holds particular significance in the advancement of innovative immunotherapeutic approaches for pancreatic cancer [7, 13, 14].

Nanomedicines offer significant advantages in co-delivering multiple components to trigger broad antitumor immune responses, serving as both passive and active delivery vehicles [15–21]. Among them, developing nanomedicines capable of specifically activating immune cells to target and eliminate tumor cells is crucial [22]. Macrophages, abundant in the tumor microenvironment (TME), play a vital role in disease progression by influencing immunometabolism [23, 24]. However, tumor cells have evolved mechanisms to evade activation and phagocytosis of macrophages via the extensive expression of anti-phagocytic molecules, such as CD47 [25, 26]. As such, enhancing both tumor cell recognition and macrophage activation/phagocytosis is essential for macrophage-based immune responses.

In recent years, Claudin18.2 (CLDN18.2) has emerged as a potential target for cancer therapy, especially for “cold” pancreatic cancer [27–29]. Additionally, CD40, a costimulatory molecule expressed on antigen-presenting cells including macrophages, when activated by agonistic anti-CD40 antibodies, induces macrophage activation and phagocytosis [30, 31]. To facilitate macrophage-mediated immunotherapy, we developed a bispecific single-chain variable fragments (scFv)-based nanoengager aimed at enhancing macrophage phagocytic activity by specifically targeting tumor cells. We genetically engineered cell membranes to obtain bispecific scFv, consisting of anti-CD40 scFv and anti-CLDN18.2 scFv. These scFv were utilized to coat a PLGA core, resulting in nanoengagers that display anti-CD40 scFv for engaging with macrophages and anti-CLDN18.2 scFv to interact with targeted tumor cells. The presence of anti-CLDN18.2 scFv on the nanoengagers enabled specific recognition of CLDN18.2-positive tumor cells by macrophages. Simultaneously, CD40-mediated treatment induced macrophage activation and antigen presentation. Subsequently, we investigated the immune responses induced by these nanoengagers and their potential for in vivo anticancer efficacy in “cold” pancreatic cancer.

## Materials and methods

### Cell lines and mice

The pancreatic tumor cell line *Kras*^*em4(LSL−G12D)*^*Trp53*^em4(R172H)^*Pdx1*^*em1(Avi−CreERT2)*^ (KPC) cell line was purchased from Shanghai Model Organisms Center and were cultured in Dulbecco’s modified Eagle medium (DMEM) with 10% fetal bovine serum (FBS), 100 U mL^− 1^ penicillin and 100 U mL^− 1^ streptomycin. HEK 293T cells were ordered from the American Type Culture Collection and also maintained in DMEM supplemented with 10% FBS, 100 U mL^− 1^ penicillin and 100 U mL^− 1^ streptomycin. KPC-CLDN18.2 cells were kindly provided by Dr. Hongkai Zhang’s lab. Humanized CD40 (hCD40) C57BL/6-*Cd40*^*em1(hCD40)Smoc*^ (6–8 weeks) mice were purchased from Shanghai Model Organisms Center (Shanghai, China). Nude mice (6 weeks) were ordered from Vital River Laboratories (Beijing, China). All animal studies were conducted in accordance with the guidelines approved by the Animal Ethics Committee of Nankai University.

### Anti-CD40/anti-CLDN18.2/OVA-overexpressing cell lines

Overexpression of anti-CD40/CLDN18.2 scFv or OVA in tumor cell lines were performed by following our previously established approach [17]. The sequences of anti-CLDN18.2 scFv (IMAB362) and OVA can be found in Table S1 and anti-CD40 scFv sequences are available upon request. Briefly, a recombinant lentivirus plasmid was constructed to express the full length of OVA, anti-CD40 or CLDN18.2 scFv on the cell surface. For anti-CD40 or anti-CLDN18.2 scFv, the scFv domain was fused to the C-terminal of IL-2 signal peptide and to the N-terminus of PDGFR transmembrane domain, generating the Lentivirus-anti-CD40 or Lentivirus-anti-CLDN18.2 scFv expression plasmids. Similarly, the sequence of full length of OVA was linked to C-terminus of the signal peptide and N-terminus of PDGFR transmembrane domain. To facilitate the sorting of transfection-positive cells, a mCherry fluorescent tag was fused to the PDGFR transmembrane sequence. The transfection of Lentivirus-anti-CD40/CLDN18.2 scFv or Lentivirus-OVA into HEK 293T cells was conducted in conjunction with packaging plasmids with using Fugene@6 (E2691, Promega) transfection reagent, following the manufacturer’s instructions. Subsequently, the medium was replaced with fresh medium after 16 h incubation, and the medium containing lentivirus was collected at 24 and 48 h post-incubation. The lentivirus-containing medium (4 mL) was then added to the target cells (i.e. KPC, KPC-CLDN18.2). The cells overexpressing anti-CD40/CLDN18.2 scFv or OVA were isolated by the sorting of mCherry fluorescence-activated cells. To examine the expression of OVA on OVA-overexpressing KPC-CLDN18.2 cells, the cells were blocked with anti-mouse CD16/32 (Biolegend, Cat. #101302) at 4 °C for 10 min, followed by stained with anti-mouse H-2kb bound to SIINFEKL-APC (Biolegend, Cat. #141605) at 4 °C for 40 min. The expression of CLDN18.2 on the CLDN18.2-overexpressing KPC was determined by stained with anti-human CLDN18.2 IgG, followed by stained with goat anti-human IgG H&L Dylight 650 antibody (abcam, ab96910). The expression of the anti-CD40/CLDN18.2 scFv on KPC cells were determined by flow cytometry and confocal laser scanning microscopy (CLSM).

### Preparation of Nano/TM, Nano/CD40, Nano/CLDN18.2 or NanoBE

The preparation of Nano/TM, Nano/CD40, Nano/CLDN18.2 and NanoBE was conducted following previously reported method [17, 32]. Briefly, KPC or anti-CD40/CLDN18.2 scFv KPC cells were collected and suspended in a hypotonic lysing buffer containing 1 mM NaHCO_3_, 0.2 mM EDTA, and 1 mM phenylmethylsulfonyl fluoride (PMSF) at 4 ^ο^C. Subsequently, the mixtures were sonicated at a power of 60 W for 15 min on ice to obtain membrane fragments. The pellet including cell debris, was discarded and the resulting supernatant was collected after centrifugation at 3200 *g* for 15 min at 4 ^ο^C. The resulting pellet was then collected following centrifugation at 16,000 *g* for 30 min at 4 ^ο^C, followed by dispersion in 200 µL of PBS containing PMSF, and stored at -80 ^ο^C.

A 50:50 poly (D,L-lactide-co-glycolide) (Mw 7000–17,000, Sigma) was used to prepare the PLGA nanoparticle cores. The mixture of PLGA in dichloromethane and 0.1 M NaHCO_3_ was sonicated on ice at a power of 200 W for 2 min using a microtip probe to generate the primary emulsion. An outer water phase consisting of 1% sodium cholate hydrate (Sigma) was added, and the mixture was sonicated at 200 W for 4 min. The resulting emulsion was then dispersed in 0.5% sodium cholate hydrate and magnetically stirred at room temperature to facilitate solvent evaporation. After centrifugation at 12,000 rpm for 10 min, the pelleted nanoparticles were washed and redispersed in PBS.

The mixture of PLGA nanoparticles and cell membrane was sonicated in a water bath sonicator, and subsequently extruded through a 400 nm polycarbonate porous membrane 20 times to obtain the cell membrane-coated PLGA nanoparticles. Specifically, Nano/TM was prepared by coating the KPC cell membrane onto PLGA nanoparticles. Nano/CD40 and Nano/CLDN18.2 were generated from anti-CD40 scFv-overexpressing KPC cells and anti-CLDN18.2 scFv-overexpressing KPC cells, respectively. NanoBE was created by coating an equivalent amount of cell membrane from both anti-CD40 scFv-overexpressing KPC cells and anti-CLDN18.2 scFv-overexpressing KPC cells. The signal intensity of mCherry in each nanoformulation was determined by flow cytometry. The protein content in each nanoformulation was examined via SDS-PAGE electrophoresis. To further examine whether the NanoBE incorporated both the anti-CD40 scFv and anti-CLDN18.2 scFv cell membrane, the cell membranes of KPC-anti-CD40 scFv and KPC-anti-CLDN18.2 scFv was first stained with DiD and DiO, respectively, followed by preparation of the NanoBE and analysis with flow cytometry.

### Cell binding assay

To evaluate the specific binding efficacy of Nano/CLDN18.2, the fluorescently labeled Nano/TM, Nano/CD40 and Nano/CLDN18.2 were prepared. A lipophilic fluorescent dye, DiD (Ex/Em = 646/663 nm; Biotium), was introduced into the oil phase during the PLGA nanoparticles preparation. KPC-CLDN18.2 or KPC tumor cells were then seeded into the 6-well plate at a density of 1 × 10^6^ cells/well. Following 12 h incubation, DiD-labeled Nano/TM or Nano/CLDN18.2 (equivalent to protein concentration, 0.5 µg/mL) were added and co-incubated with the cells for 6 h. Any unbound particles were then removed by washing the cells with PBS for three times. The binding efficacy was measured using flow cytometry (BD), and the data were analyzed using FlowJo X software.

### Macrophage isolation and activation

Bone marrow-derived macrophages (BMMs) were isolated according to a previously published protocol [33]. Briefly, the femurs were harvested from each leg of CD40-humanized transgenic mouse. Then the marrow was flushed from the femurs using a syringe containing sterile PBS. Afterward, the marrow was centrifuged, and the erythrocytes were removed using an ammonium-chloride-potassium (ACK) lysis buffer (Solarbio). The cells were subsequently cultured in Roswell Park Memorial Institute (RPMI)-1640 supplemented 10% FBS and 10 ng/mL murine M-CSF (315-02, Peprotech). On the third day of culture, fresh medium containing 10 ng/mL murine M-CSF were replaced.

For the macrophage activation assay, BMMs collected on Day 6 were seeded in 6-well plates at a density of 8 × 10^5^ cells/well. After16 h treatment with PBS, Nano/TM or Nano/CD40 (equivalent to protein concentration, 0.3 µg/mL), the BMMs were collected and incubated with anti-mouse CD16/32 (Biolegend, Cat. #101302) at 4 °C for 10 min. Subsequently, they were stained with anti-mouse F4/80-PE/Cy7 (Biolegend, Cat. #123113), CD80-BV711 (Biolegend, Cat. #123147) and anti-mouse CD86-BV650 (Biolegend, Cat. #105036) at 4 °C for 40 min. The resulting cells were analyzed by flow cytometry (BD LSRFortessa X-20), and the data analysis was conducted using FlowJo X software.

### Phagocytosis assay

For the phagocytosis assay, BMMs collected on day 6 were labeled with a fluorescent dye, DiD, at a working concentration for 15 min at 37 ^ο^C. The excess DiD was removed by washing the cells with PBS for three times. DiD-labeled BMMs were seeded into the 6-well plates at a density of 1 × 10^6^ cells/well. Similarly, KPC or KPC-CLDN18.2 cells were labeled with another fluorescent dye, DiO (Ex/Em = 484/501 nm; Biotium), and co-cultured with DiD-labeled BMMs at a density of 1 × 10^5^ cells/well. Following 6 h treatment with PBS, Nano/TM, Nano/CD40, Nano/CLDN18.2 or NanoBE (equivalent to protein concentration, 0.3 µg/mL), the cells were harvested and the fluorescent intensity of DiO-labeled KPC or KPC-CLDN18.2 cells within the DiD-labled BMMs was determined using flow cytometry. The data were analyzed by FlowJo X software.

### Antigen presentation

OVA-overexpressing KPC-CLDN18.2 tumor cells were seeded at a density of 1 × 10^5^ cells/well and co-cultured with BMMs at a density of 5 × 10^5^ cells/well in the 6-well plates for 12 h. This co-culture was performed in the presence of PBS alone, Nano/TM, Nano/CD40, Nano/CLDN18.2 or NanoBE (equivalent to protein concentration, 0.3 µg/mL). After 12 h incubation, the cells were blocked with anti-mouse CD16/32 at 4 °C for 10 min. Subsequently, they were stained with anti-F4/80-PE/Cy7 and anti-mouse H-2kb bound to SIINFEKL-APC (Biolegend, Cat. #141605) at 4 °C for 40 min before being subjected to flow cytometry analysis. The data analysis was performed using FlowJo X software.

### T-cell priming

For T-cell priming, KPC-CLDN18.2 tumor cells were seeded and co-cultured with BMMs at a ratio of 1:5 in the presence of PBS alone, Nano/TM, Nano/CD40, Nano/CLDN18.2 or NanoBE. After 12 h incubation, T cells were isolated from C57BL/6 mice using the mouse CD3^+^T cell isolation kit (Selleck) following the manufacturer’s protocols. Subsequently, the isolated T cells were co-cultured with pre-treated BMMs at a ratio of 10:1. After 24 h of incubation, the supernatant was collected to measure the cytokine IFN-γ using ELISA according to the manufacturer’s protocols (Elabscience).

### Biodistribution assay

To observe the biodistribution, DiR (Ex/Em = 750/780 nm; Biotium)-labeled Nano/TM, Nano/CD40, Nano/CLDN18.2 and NanoBE were prepared. 200 µl of DiR-labeled Nano/TM, Nano/CD40, Nano/CLDN18.2 or NanoBE (equivalent to DiR concentration, 150 µg/mL) were intravenously injected into CD40-humanized transgenic mice. At 1, 2, 4, 8, 12, 24 and 48 h post-injection, the mice were anesthetized for fluorescence imaging using a Xenogen IVIS Lumina II imaging system. At 48 h post-injection, the major organs including livers, spleens, hearts, lungs and kidneys were collected for ex vivo fluorescence imaging.

### Antitumor efficacy

Male CD40-humanized transgenic mouse (7 weeks) were subcutaneously injected with 1 × 10^6^ KPC-CLDN18.2 tumor cells to evaluate the antitumor therapeutic efficacy of each nanoformulation. The mice were intravenously injected with PBS, Nano/TM, Nano/CD40, Nano/CLDN18.2 or NanoBE (equivalent to protein amount, 30 µg) at Day 16, 18 and 20. The tumor volume was continuously monitored and calculated using the formula: length × width^2^/2. The experimental endpoint of survival analysis was determined as either death or reaching a tumor volume of 1500 mm^3^.

### Tumor-infiltrating T lymphocytes analysis and safety assessment

The infiltration of T lymphocytes within the tumor microenvironment was evaluated according to previously published protocol [34]. Briefly, CD40-humanized transgenic mice bearing KPC-CLDN18.2 cells were euthanized on Day 34, after undergoing three rounds of treatment with PBS, Nano/TM, Nano/CD40, Nano/CLDN18.2 or NanoBE on Day 16, 18 and 20. Tumors were harvested and mechanically disrupted, followed by digested with dnase I (Solarbio), dispase II (Solarbio) and collagenase IV (Solarbio). The resulting cells were filtered with 70 μm cell strainer (Biosharp) to obtain single-cell suspensions. These cells were then stained with a Viability dye, blocked using anti-mouse CD16/32, and subsequently incubated with anti-mouse CD45 (Biolegend, Cat. #103108), anti-mouse CD3 (Biolegend, Cat. #100218), anti-mouse CD4 (Biolegend, Cat. #100428), anti-mouse CD8 (Biolegend, Cat. #100752). For intracellular staining, the cells were fixed and permeabilized using the True-Nuclear Transcription Factor Buffer Set (Biolegend, Cat. #424401) before being stained with anti-mouse IFN-γ (Biolegend, Cat. #505807). Flow cytometry was performed to analyze the samples, and the data were analyzed using FlowJo X software. Meanwhile, the livers of different groups were collected, and liver sections were subjected to the Hematoxylin and Eosin (H&E) staining to evaluate the hepatotoxicity under an optical microscope. The cell apoptosis in tumor tissue was determined with a TUNEL assay kit (Elabsceince). Representative images were captured with a confocal microscopy (Zeiss) and quantified using ImageJ. The tumor sections were subjected to immunohistochemical staining for CD8^+^ T cells infiltrated in the tumor tissues, according to the manufacturer’s protocols (Abcam, Elabscience). Masson’s trichrome staining was performed to evaluate the expression of collagen in tumor tissues (solarbio). The immunohistochemical and Masson’s trichrome staining were visualized using an optical microscope and quantified using ImageJ.

### Statistical analysis

One-way ANOVA or two-way ANOVA was used for multiple comparisons. Two-tailed t-test was used for two-group comparisons. Survival curves were analyzed with Kaplan-Meier method and compared by the log-rank test. All the statistical analyses were carried out with Prism (v9.0; GraphPad Software). A p-value less than 0.05 was considered statically significant.

## Results

### Design and characterization of NanoBE

**Fig. 1:**
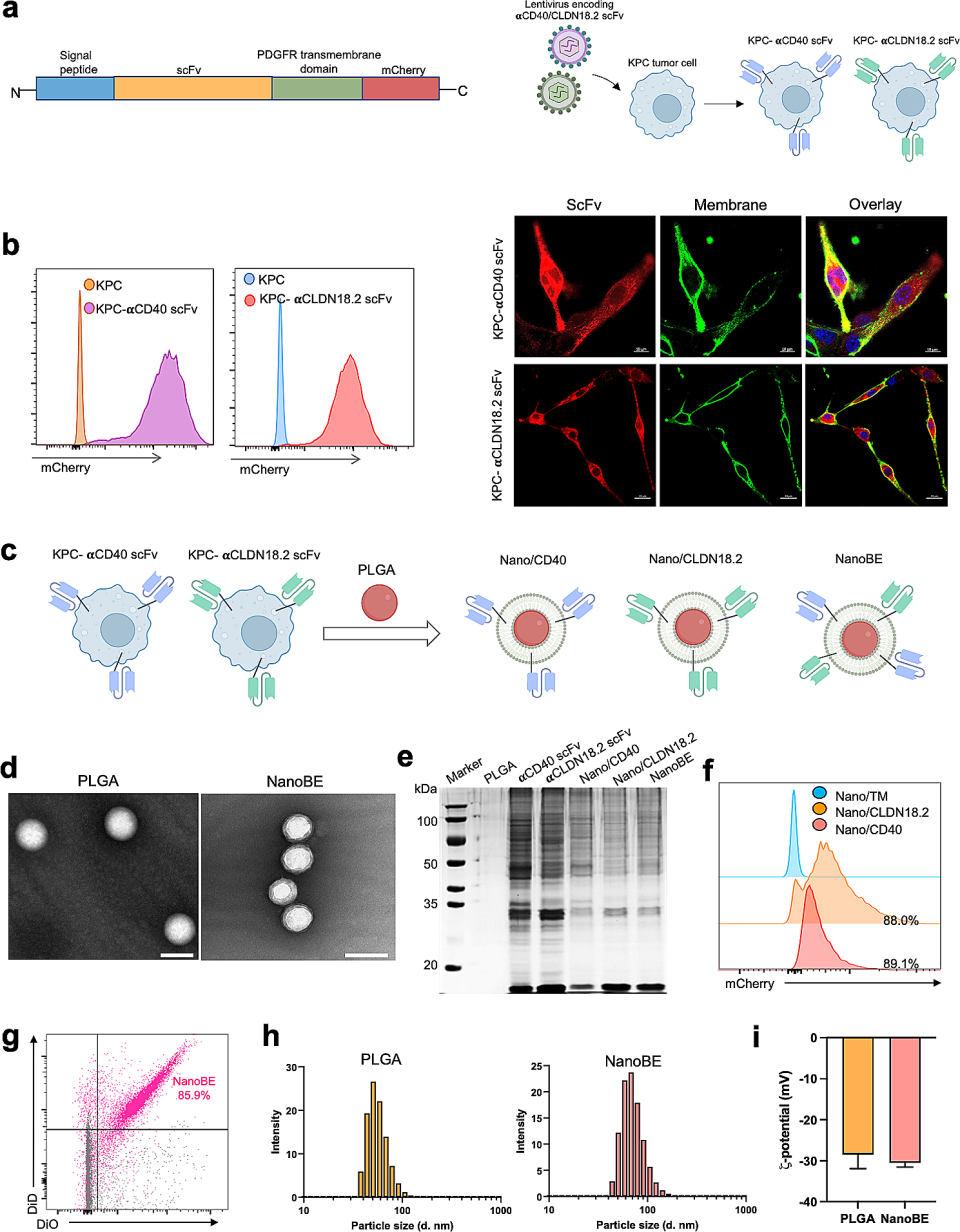
Design, preparation and characterization of NanoBE. (**a**) Schematic illustration of recombinant lentivirus LV-𝛂CD40/CLDN18.2 scFv plasmid. The scFv sequence was fused to the C-terminus of the signal peptide and the N-terminus of PDGFR transmembrane domain. The cells were transfected with the constructed recombinant lentivirus. (**b**) Flow cytometry analysis (left) and confocal imaging (right) of mCherry-positive cells showed the 𝛂CD40 scFv and 𝛂CLDN18.2 scFv expression. (**c**) Preparation of Nano/CD40, Nano/CLDN18.2 and NanoBE. Nano/CD40 and Nano/CLDN18.2 was prepared by coating cell membrane from KPC-𝛂CD40 scFv and KPC-𝛂CLDN18.2 scFv onto PLGA nanoparticle core, respectively. NanoBE was prepared using cell membrane derived from both KPC-𝛂CD40 scFv and KPC- 𝛂CLDN18.2 scFv. (**d**) TEM images of PLGA alone and NanoBE. Scale bar = 100 nm. (**e**) SDS-PAGE protein analysis of PLGA, cell membrane isolated from KPC-𝛂CD40/𝛂CLDN18.2 scFv, Nano/CD40, Nano/CLDN18.2, and NanoBE. (**f**) Flow cytometry analysis of the mCherry signal on Nano/CLDN18.2 and Nano/CD40. (**g**) Flow cytometry analysis of NanoBE containing both DiD-labeled KPC-𝛂CD40 scFv and DiO-KPC-𝛂CLDN18.2 scFv membrane. Gray, the particles without cell membrane; pink, the particles with both cell membrane. (**h**) Particle size and (**i**) 𝛇-potentials of PLGA alone and NanoBE

In our previous study, we developed an antibody-anchored membrane technology through the recombination of scFv into lentivirus plasmids, allowing for displaying of scFv on the surface of cell membrane [17]. The recombinant lentivirus plasmid comprises four key components: IL-2 signal peptide, scFv, platelet-derived growth factor receptor (PDGFR) transmembrane domain, and mCherry (Fig. 1a). IL-2 and PDGFR serve to facilitate the overexpression of scFv on the cell membrane and ensure the correct orientation of scFv on the cell membrane surface, respectively. The mCherry fluorescence signal aids in the sorting of successfully transfected cells. Notably, CD40 is a well-known costimulatory molecule expressed on macrophages [35]. Additionally, CLDN18.2 demonstrates high expression levels in clinical patients with pancreatic cancer. To align our research with clinical relevance, we thus selected anti-CD40 scFv and anti-CLDN18.2 scFv sequences that enable recognize macrophages and pancreatic cancer in human. In a typical nanoengager construction, anti-CD40 scFv and anti-CLDN18.2 scFv were firstly incorporated into the cell membrane using our antibody-anchored membrane technology. Flow cytometry analysis of mCherry-sorted cells confirmed the successful anchoring of anti-CD40 scFv (αCD40 scFv) and anti-CLDN18.2 (αCLDN18.2 scFv) into KPC pancreatic cancer cells, respectively (Fig. 1b, left). Additionally, confocal images exhibited the co-localization of the mCherry signal and cell membrane, indicating the antibodies fragments can be displayed on the cell membrane (Fig. 1b, right). Subsequently, the scFv-anchored membrane nanomedicines were prepared by employing PLGA nanoparticles as the core and extracted cell membrane as the surface coating through a membrane extruded method. In other words, Nano/CD40 and Nano/CLDN18.2 was produced by coating the cell membrane derived from the KPC-αCD40 scFv and KPC-αCLDN18.2 scFv onto the PLGA nanoparticles, respectively. Furthermore, bispecific nanoengagers (NanoBE) were also created by coating with an equivalent mixture of cell membrane from both KPC-αCD40 scFv and KPC-αCLDN18.2 scFv (Fig. 1c). Transmission electron microscopy (TEM) images provided evidence that the cell membrane was effectively coated onto the PLGA nanoparticle cores (Fig. 1d). SDS-PAGE analysis demonstrated that the protein components extracted from NanoBE included both membrane proteins from the KPC-αCD40 scFv and KPC-αCLDN18.2 scFv-engineered cells, confirming that NanoBE contained membrane proteins from two different engineered cells (Fig. 1e). Moreover, 88.0% of the Nano/CLDN18.2 and 89.1% of Nano/CD40 showed significant mCherry signal, suggesting the successful coating of αCLDN18.2 and αCD40 scFv onto the corresponding nanoformulation (Fig. 1f). Flow cytometry analysis further conformed that 85.9% of the NanoBE particles exhibited both membranes containing KPC-αCD40 scFv and KPC-αCLDN18.2 scFv (Fig. 1g). By employing dynamic light scattering (DLS) analysis, we observed a uniform size distribution and a slight increase in size after coating with cell membrane (Fig. 1h). Simultaneously, a slight reduction in the ζ-potential was observed after coating with cell membrane (Fig. 1i**)**. Additionally, the TEM image, size distribution and ζ-potential of Nano/CD40 and Nano/CLDN18.2 were not significantly changed compared to those of NanoBE.

### Bispecific crosslinking between macrophages and tumor cells

**Fig. 2:**
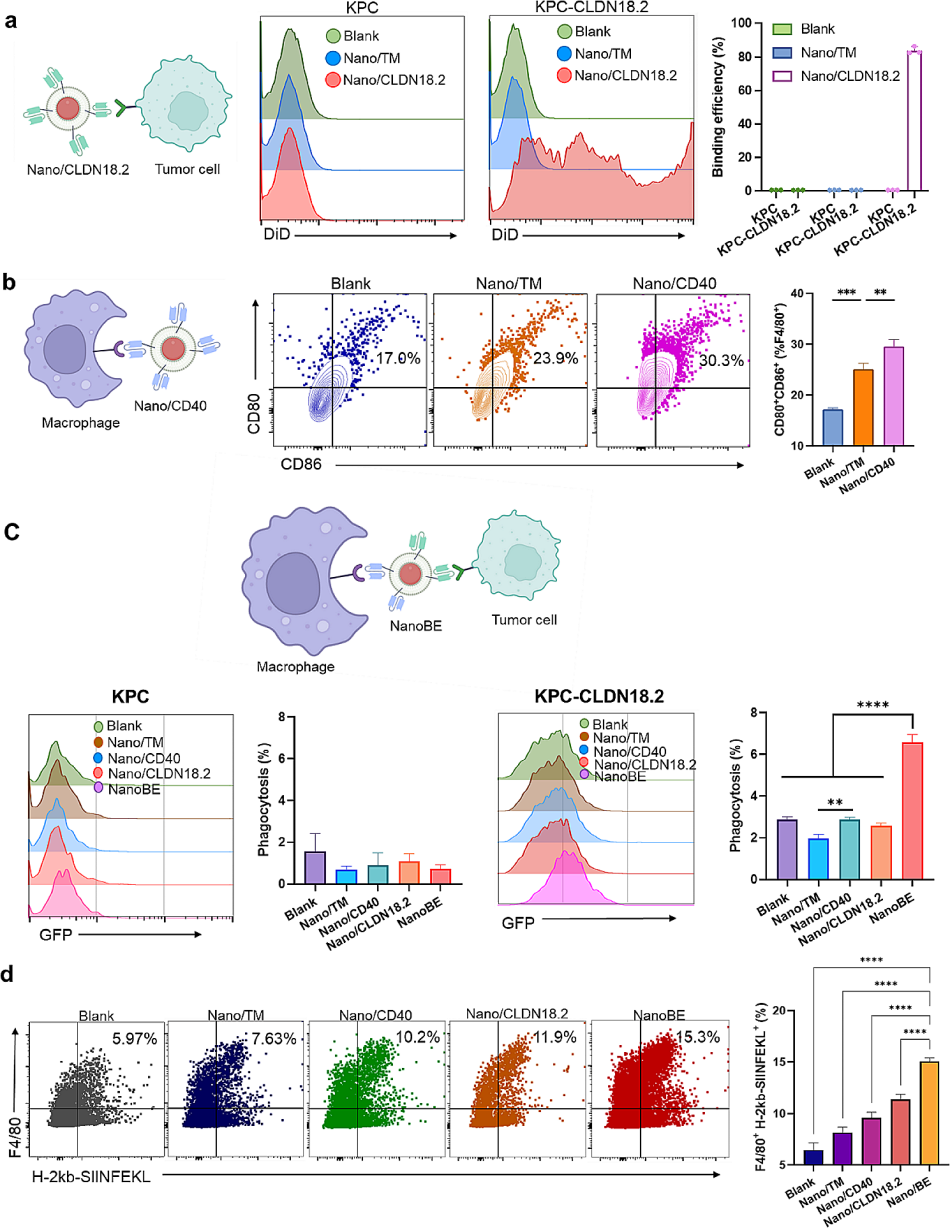
NanoBE facilitated bi-specific engagement of tumor and macrophages. **a**) Binding affinity of Nano/CLDN18.2 to KPC and KPC-CLDN18.2 tumor cells. After particle binding, representative flow cytometry results (middle) and quantitative analysis (right) of tumor cells were shown. **b**) Flow cytometry analysis of BMM activation induced by Nano/TM and Nano/CD40. **c**) Flow cytometry analysis showing phagocytosis of KPC or KPC-CLDN18.2 cells by BMMs treated with Nano/TM, Nano/CD40, Nano/CLDN18.2 or NanoBE. **d**) Antigen presentation of BMMs after phagocytosis of OVA-expressing KPC-CLDN18.2 cells treated with Nano/TM, Nano/CD40, Nano/CLDN18.2 or NanoBE. Statistical analysis between the indicated groups was conducted using one-way ANOVA with Tukey’s multiple comparisons test (b-d). ^**^p < 0.01, ^***^p < 0.001, ^****^p < 0.0001

CLDN18.2 is typically highly expressed in human-derived pancreatic cancer cells, where mouse-derived pancreatic cancer cell lines with high CLDN18.2 expression is rare. To facilitate immunotherapy in a tumor-bearing mouse model, it becomes essential to establish a mouse pancreatic cancer cell line with a stable, high CLDN18.2 expression. Thus, we first construct KPC pancreatic cancer cells with stable, high CLDN18.2 expression, as confirmed by flow cytometry analysis of the sorted KPC cells (Fig. S1a). The expression of human CLDN18.2 on KPC-CLDN18.2 cells was further confirmed by staining with anti-human CLDN18.2 antibody and analyzed by flow cytometry (Fig. S1b). To evaluate the effectiveness of NanoBE engagement, we first conducted the separated studies on the specific binding of Nano/CLDN18.2 with tumor cells and the macrophage activation induced by Nano/CD40, respectively. As illustrated in Fig. 2a, Nano/CLDN18.2 significantly enhanced the binding affinity of nanoparticles to CLDN18.2-overexpressing tumor cells. In contrast, a control nanomedicine, Nano/TM, containing KPC cell membrane but lacking scFv transfection, did not exhibit this effect. Flow cytometry analysis demonstrated that over 80% of CLDN18.2-overexpressing tumor cells were efficiently bound to Nano/CLDN 18.2. However, Nano/CLDN18.2 did not exhibit enhanced binding affinity to CLDN 18.2-negative tumor cells, indicating that the binding efficiency was mediated by the CLDN18.2 receptors. Subsequently, we investigated whether Nano/CD40 acts as an agonist to trigger macrophage activation, thereby enhancing their phagocytic activity. As Nano/CD40 or NanoBE incorporated a humanized CD40 scFv, bone marrow-derived macrophages (BMMs) were isolated from CD40-humanized transgenic mouse. After treatment with Nano/CD40, the expression of costimulatory molecules CD80/CD86 on the BMMs significantly increased compared to that of Nano/TM (Fig. 2b), indicating that Nano/CD40 promotes the recognition and activation of BMMs.

We next examined whether NanoBE improved phagocytosis of tumor cells by macrophages. BMMs were isolated from CD40-humanized transgenic mouse, and DiO-labeled KPC cells were co-cultured with DiD-labeled BMMs at a ratio of 1:10 (tumor cells:BMMs) for 6 h. Phagocytosis of BMMs was determined by analyzing the DiO fluorescence intensity after gating for DiD signal. The results demonstrated that BMMs treated with NanoBE exhibited the highest phagocytic capacity (6.58%) toward KPC-CLDN18.2 cells, significantly surpassing Nano/TM, Nano/CD40 and Nano/CLDN18.2. In contrast, NanoBE treatment did not enhance the phagocytosis of tumor cells in KPC cells (without CLDN18.2 overexpression) by BMMs. This suggest that the increased phagocytosis induced by NanoBE was highly dependent on the CLDN18.2 receptor expression level (Fig. 2c). Inspired by the receptor-dependent enhancement of phagocytosis, we further investigated whether receptor-mediated phagocytosis could facilitate antigen processing and presentation by BMMs. To explore this, we employed a model antigen, ovalbumin (OVA), and display it on the cell membrane surface using the same antibody-anchored membrane technology. Following the sorting of OVA-overexpressed CLDN18.2-positive tumor cells, 86.2% of the KPC-CLDN18.2-OVA cells demonstrated the expression of the OVA-peptide SIINFEKL (Fig. S1c, d). OVA antigen presentation was subsequently checked by co-culturing BMMs with OVA-overexpressed CLDN18.2-postive tumor cells. Compared to treatment with Nano/TM, Nano/CD40 and Nano/CLDN18.2, the capacity of BMMs treated with NanoBE to present the OVA antigen increased by 200%, 150% and 129%, as evidenced by the presence of the OVA peptide epitope on the major histocompatibility complex class I (MHC-I) complex (H-2 kb-SIINFEKL) (Fig. 2d). Thus, the enhanced phagocytosis induced by NanoBE resulted in a significantly higher cross-presentation of OVA peptide on the MHC-class I complex on the surface of BMMs. Following nanoengager-mediated macrophage activation and antigen presentation, we subsequently examined whether T cell priming could be induced. T cells were co-cultured with BMMs pre-treated with KPC-CLDN18.2 cells using various nanoformulations. The NanoBE treatment resulted in a more significant production of IFN-γ, exhibiting an increase of approximately 4-fold (Fig. S2).

### Tumor targeting and in vivo biodistribution

**Fig. 3:**
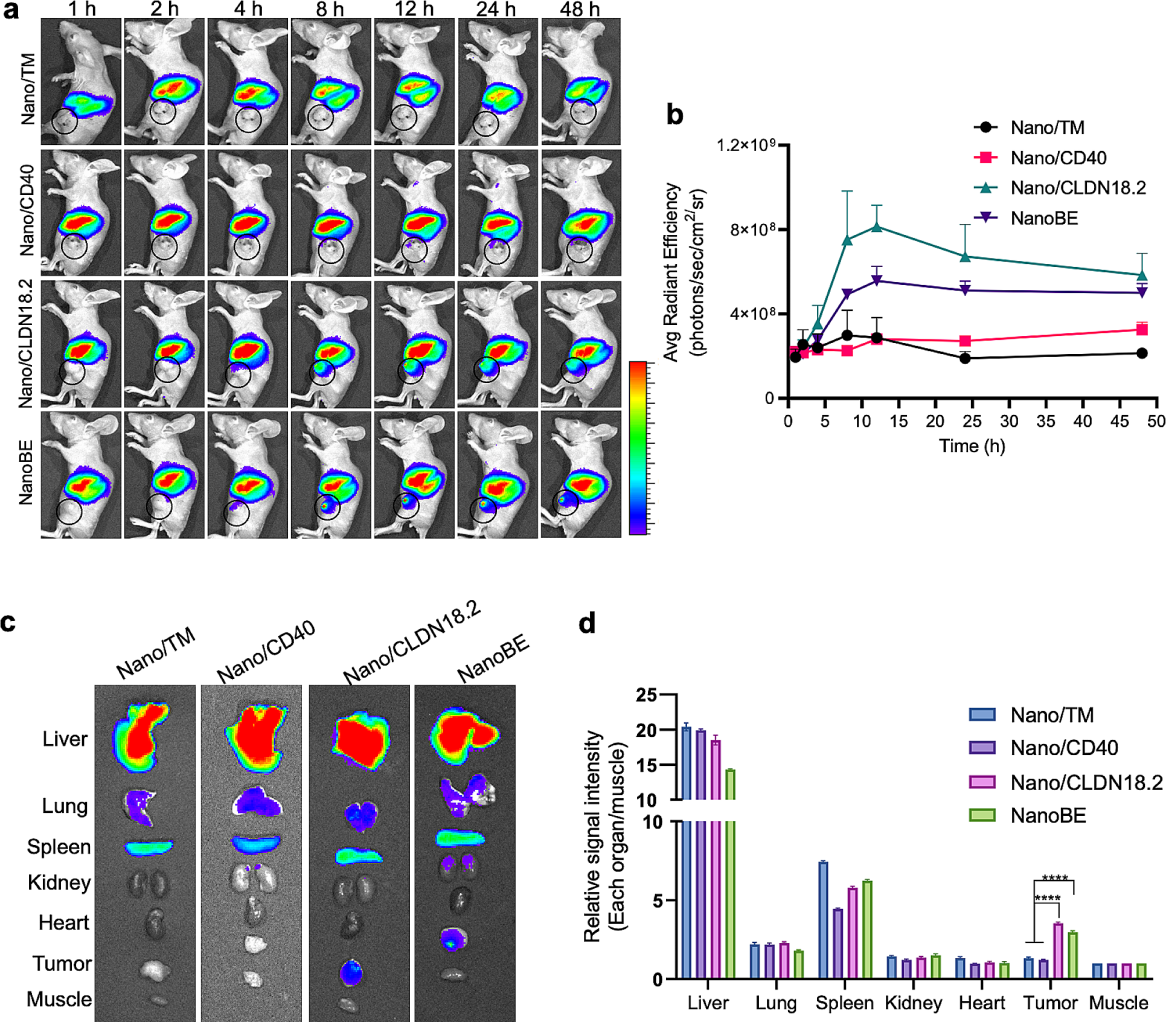
Tumor targeting and biodistribution. (**a**) Representative in vivo fluorescence imaging of biodistribution at the indicated time points after i.v. injection of Nano/TM, Nano/CD40, Nano/CLDN18.2 and NanoBE. Tumors were indicated by black circle. (**b**) Mean signal intensity of tumors following administration of the particles at the indicated time points. (**c**) Representative ex vivo fluorescent images of major organs at 48 h post-injection. (**d**) Relative signal intensity of each organ normalized to that of the muscle at 48 h post-injection. Statistical analysis between the indicated groups was performed using two-way ANOVA with Tukey’s multiple comparisons test. ^****^p < 0.0001

It is necessary for NanoBE to possess superior tumor-accumulating capacities to facilitate the phagocytosis of tumor cells by macrophages, thereby triggering subsequent anti-tumor immune responses. To visualize the biodistribution of various nanoformulations in nude mice bearing CLDN18.2-overexpressing KPC tumor cells, we loaded the hydrophobic fluorescence dye DiR into the PLGA core of each formulation. Following systematic administration via the tail vein, we conducted in vivo imaging of the mice at specified time points using a Xenogen IVIS imaging system. Remarkably, unlike Nano/TM and Nano/CD40, DiR-loaded Nano/CLDN18.2 and NanoBE exhibited enhanced tumor accumulation and prolonged retention over time (Fig. 3a, circle). In both Nano/CLDN18.2 and NanoBE groups, we observed a gradual increase in tumor accumulation over time, with maximal accumulation at 12 h. Quantification analysis of signal intensity from the tumors demonstrated that Nano/CLDN18.2 and NanoBE exhibited significantly higher accumulation compared to Nano/TM and Nano/CD40 (Fig. 3b), highlighting the pivotal role of anti-CLDN18.2 scFv in tumor targeting. To further evaluate the biodistribution of these nanomedicines, we harvested major organs and tumors for ex vivo fluorescent imaging at 48 h-post injection. Tumors collected from both the Nano/CLDN18.2 and NanoBE groups showed approximately a 2.3-fold higher fluorescent signal compared to those from the Nano/TM and Nano/CD40 groups, as illustrated in Fig. 3c and d. All nanoformulations exhibited higher and comparable distribution in the liver, spleen, and lung, with negligible distribution observed in the kidney, heart, and muscle. The enhanced tumor accumulation of Nano/CLDN18.2 and NanoBE could be attributed to the modification with anti-CLDN18.2 scFv, which facilitated binding to CLDN18.2 on the tumor cells.

### In vivo anti-tumor efficacy

**Fig. 4:**
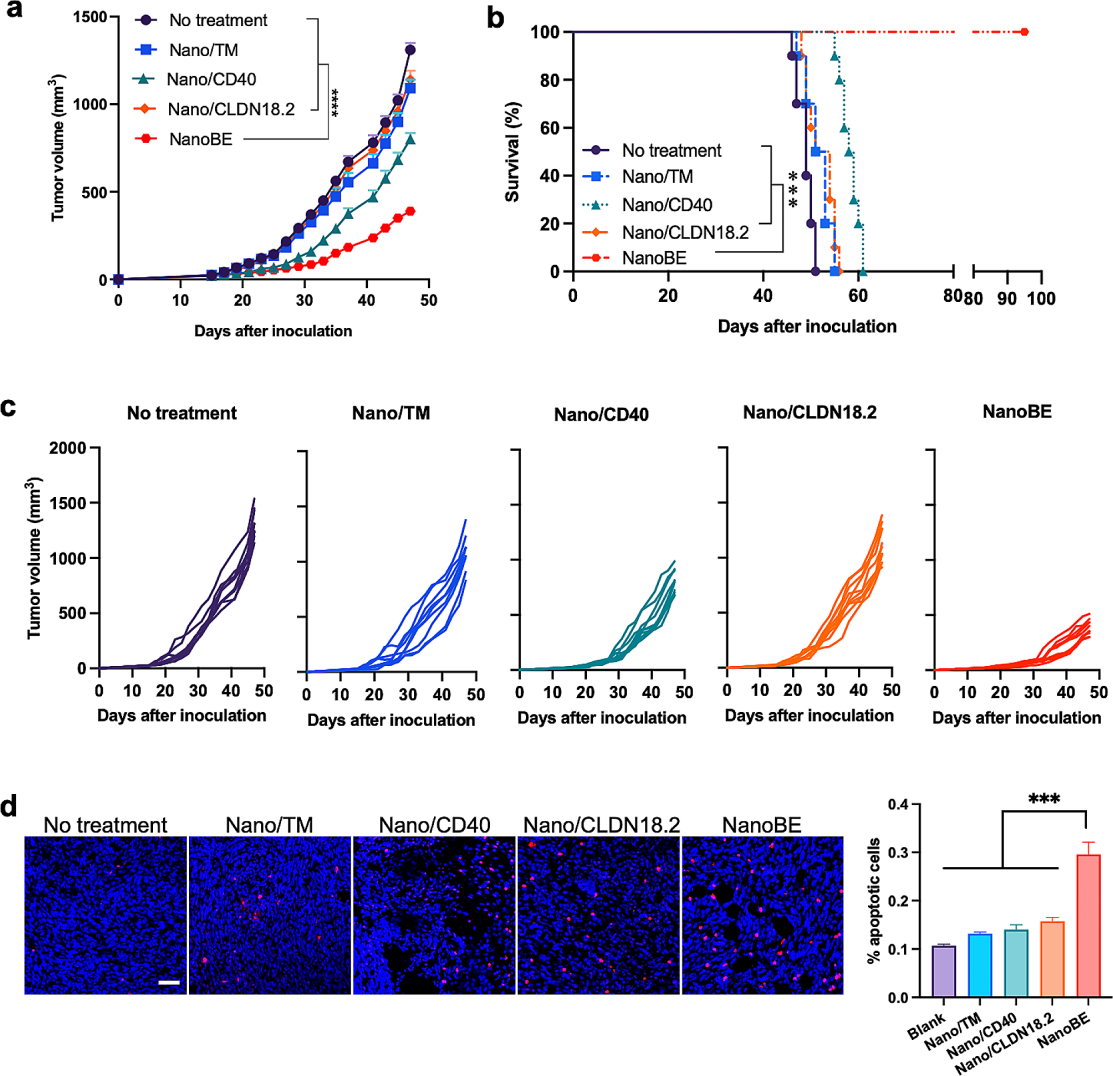
In vivo antitumor efficacy of NanoBE. (**a**) Mean tumor growth curves of KPC tumors with different treatments (*n* = 10 each group). Data are presented as mean ± SEM. (**b**) Kaplan-Meier survival curves of mice in the different treatment groups (*n* = 10 each group). (**c**) Individual tumor growth curves of KPC tumors in each treatment group (*n* = 10 each group). (**d**) TUNEL staining of apoptotic cells in the KPC tumors. (*n* = 3 independent mice in different treatment groups) Green, TUNEL; Blue, nucleus. Scale bar = 50 μm. Statistical analysis between the indicated groups was performed using two-way (a) or log rank (Mantel-Cox) test (b) or one-way ANOVA with Tukey’s multiple comparisons test (d). ^***^p < 0.001, ^****^p < 0.0001

Next, we sought to investigate the in vivo anti-tumor efficacy of NanoBE. Since macrophage inducers (i.e., Nano/CD40) contain humanized CD40 scFv, we established CLDN18.2-overexpressing KPC subcutaneous tumor models in CD40-humanized transgenic mice. These mice were then randomly assigned to five groups: untreated, Nano/TM, Nano/CD40, Nano/CLDN18.2 and NanoBE. Compared to the untreated group, neither Nano/TM nor Nano/CLDN18.2 showed statistically significant changes in tumor growth (Fig. 4a, c). In contrast, Nano/CD40 exhibited moderate anti-tumor efficacy. Importantly, NanoBE demonstrated a significant inhibition of tumor growth compared to Nano/CLDN18.2 or Nano/CD40 alone, highlighting the remarkable synergistic effect of nanoengagers through the crosslinking between macrophages and tumor cells. For survival analysis, treatment with Nano/TM and Nano/CLDN18.2 increased the median survival from 49 days in the PBS treatment group to 52 and 52.5 days, respectively. Treatment with Nano/CD40 slightly extended the median survival to 58 days. In particular, treatment with NanoBE resulted in the most significant tumor growth inhibition, leading to a survival extension of over 100 days for all mice (Fig. 4b).

**Fig. 5:**
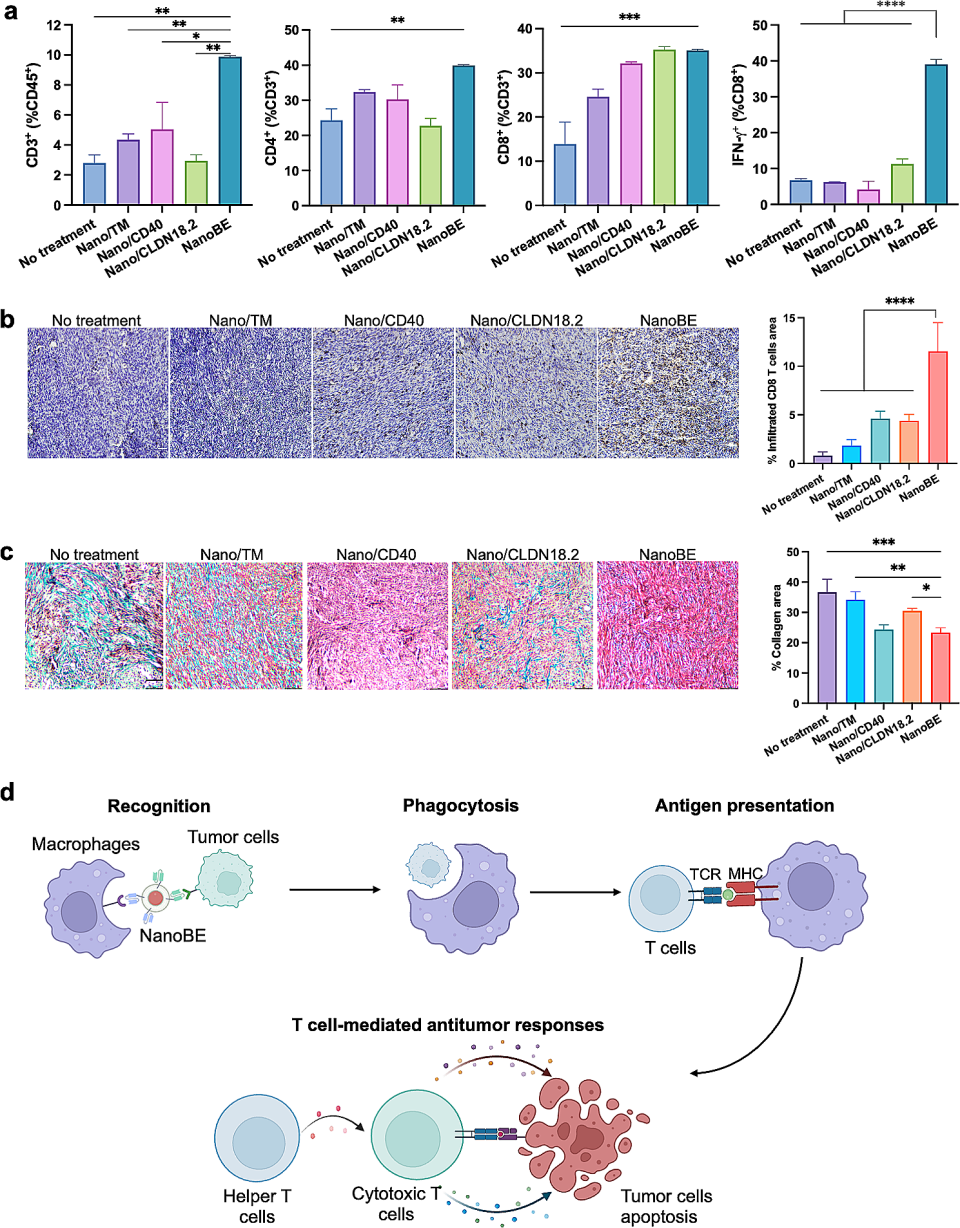
Remodeling of tumor microenvironment in KPC mouse model. (**a**) Flow cytometry analysis of CD45^+^CD3^+^ T cells ratio in total CD45^+^ cells, CD45^+^CD3^+^CD4^+^ T cells ratio in total T cells (CD45^+^CD3^+^), CD45^+^CD3^+^CD8^+^ T cells ratio in total T cells (CD45^+^CD3^+^), and CD45^+^CD3^+^CD8^+^IFN-𝛄^+^ T cells in total CD8^+^ T cells (CD45^+^CD3^+^CD8^+^) (*n* = 3 independent mice in different treatment groups) Data are shown as mean ± SEM. (**b**) IHC staining and quantitative analysis of infiltrated CD8^+^ T cells in the tumors. (*n* = 5 independent mice in different treatment groups) Data are shown as mean ± SD. Scale bar = 100 μm. (**c**) Masson’s Trichrome staining of the KPC tumors. Green, deposited collagen; red, muscle fibers. Scale bar = 100 μm. (*n* = 3 independent mice in different treatment groups) Data are shown as mean ± SD. (**d**) Schematic illustration of proposed action mechanism of NanoBE. NanoBE facilitates the recognition of tumor cells and enables the engagement of phagocytosis by macrophages. T cell-mediated anti-tumor immune responses can be elicited as the results of antigen presentation by macrophages. Created with BioRender.com. Statistical analysis between the indicated groups was performed using one-way ANOVA with Tukey’s multiple comparisons test. ^*^p < 0.05, ^**^p < 0.01, ^***^p < 0.001, ^****^p < 0.0001

To elucidate the reasons behind tumor inhibition, we conducted a TUNEL assay to measure cell apoptosis in isolated tumor tissues after the treatments. Compared to the other groups, NanoBE treatment significantly increased the apoptosis of tumor cells (Fig. 4d), consistent with its tumor inhibition ability. We further explored whether effector T cells played a role in the mechanisms underlying tumor cell apoptosis induced by NanoBE. Macrophages are central regulators in T cell functions and are involved in each step of the process, including initiating the events leading to T cell activation. Modulating costimulatory molecules such as CD40 in macrophages can alter TME conducive to T cell activity. As such, we first assessed effector T-cell infiltration into the TME using flow cytometry after the treatments. The gating strategy for the cells is illustrated in Fig. S3. In a typical analysis (Fig. 5a), NanoBE treatment resulted in the highest percentages of infiltrating CD3^+^ T cells compared to the other treatments. The percentage of CD3^+^ T cells in tumors treated with NanoBE increased by 2-fold and 3.3-fold compared to those treated with Nano/CD40 and Nano/CLDN18.2, respectively. Additionally, NanoBE treatment elevated the levels of infiltrating CD4^+^ T cells in tumors compared to the other treatments. All treatments led to increased tumor infiltrating CD8^+^ T cells, cytotoxic T lymphocytes, in the TME. Most importantly, mice treated with NanoBE exhibited significantly higher expression of IFN-γ in CD8^+^ T cells, indicating increased activity of cytotoxic T lymphocytes as a result of NanoBE treatment. Immunohistochemistry data further supported these findings, demonstrating a significant enhancement of infiltrated cytotoxic T cells in the KPC TME following NanoBE treatment compared to the other treatments (Fig. 5b). Additionally, therapeutics based on anti-CD40 agonistic antibodies typically result in serious liver toxicity [36–38]. To assess liver toxicity, the isolated livers from different treatment group were examined using H&E staining. The results illustrated that no significant morphology changes were observed in the livers from each treatment group, suggesting that there is no significant hepatoxicity upon treatment (Fig. S4). The dense stromal compartment in pancreatic cancer contributes to an immunosuppressive tumor microenvironment that limits the active infiltration of immune cells, a distinguished characteristic of this cancer type. It has been reported that CD40 activation can reverse this immunosuppressive microenvironment by facilitating the depletion of tumor stroma [30]. The degradation of the extracellular matrix plays a crucial role in remodeling T cell antitumor immunity in the tumor microenvironment. Here, we observed a significant decrease in collagen expression in tumors treated with Nano/CD40 or NanoBE (Fig. 5c), indicating that CD40 agonists may affect tumor stroma. Together, we propose the potential underlying mechanism of NanoBE as follows (Fig. 5d): NanoBE facilitates the recognition of tumor cells, and enables the engagement of phagocytosis by macrophages, resulting in elevated tumor cell-derived antigen presentation by macrophages. This antigen presentation, in turn, induces T cell-mediated anti-tumor responses, including the infiltration of cytotoxic T cells into solid tumor tissue, activation of cytotoxic T cells and T helper cells, and tumor cell apoptosis triggered by cytokines released from cytotoxic T cells.

## Discussion

Pancreatic cancer stands out as one of the most lethal carcinomas [1]. Despite advancements in cancer immunotherapy, no significant improvement in survival rates of pancreatic cancer has been demonstrated [7]. While various monoclonal antibodies have been developed to target inhibitory or stimulatory receptors on immune cells and enhance antitumor immune responses, only a minority of pancreatic cancer patients have benefits from monoclonal antibodies therapy [8, 9, 39]. This limited success is largely attributed to the immunosuppressive microenvironment typical of pancreatic cancer [10–12, 40, 41]. Bispecific antibodies have emerged as a promising alternative to monoclonal antibodies, facilitating interactions between immune effector cells and tumor cells [42]. However, the challenges in construction and acquirement of bispecific antibodies pose significant hurdles [43–45]. Recent developed nanoengagers, composed of multivalent bispecific antibody, represents a new targeted, nanomaterial-immunotherapy platform to stimulate innate and adaptive immunity and promote a universal antitumor response. While previous studies demonstrated the efficacy of nanoengagers in boosting antitumor responses through the conjugation of two antibodies onto nanoparticle surfaces, this strategy faces limitations such as restricted surface functional groups on nanoparticles, multiple modification procedures and high costs associated with expensive antibody. In this study, we present an antibody-anchored membrane technology through the recombination of scFv into lentivirus plasmids, allowing for the direct display of scFv on the surface of cell membrane. Unlike the limitation of bispecific antibodies, which are typically restricted to incorporating only two antibodies, our approach allows for the straightforward amplification of cell membranes containing various scFv, providing a versatile platform for the efficient production of nanoengagers tailored to specific requirements. For instance, our nanoengager system is capable of simultaneously displaying multiple antibodies, including blockade antibodies, targeting antibodies and agonistic antibodies.

Additionally, our technology’s advantage lies in the anchoring of the required scFv antibody into the cell membrane. As the most commonly used approach, chemical conjugation may lead to the excessive decoration of membrane proteins, potentially compromising the antigenic activities of these proteins. Furthermore, the poor reproducibility of chemical conjugation poses a significant obstacle to subsequent industrialization efforts. In contrast, genetic engineering enables stable and specific antibody expression and retention on the cell surface, ensuring that the antibodies retain their biological functions.

The nanoengagers elucidated in this study effectively augment the recognition and phagocytosis of tumor cells by macrophages. Furthermore, the nanoengagers serve as triggers, promoting the activation of macrophages and subsequent antigen processing and presentation. These combined benefits have the potential to remodel immunosuppressive tumor microenvironment, resulting in an increased infiltration of effector T cells into the tumor tissues. Consequently, a significant improvement in the antitumor efficacy against highly aggressive “cold” pancreatic cancer is observed. It is noteworthy that, the two scFv sequences (i.e., anti-CD40 scFv and anti-CLDN18.2 scFv) possess the ability to recognize both human macrophages and pancreatic cancer cells. To enable immunotherapy in a mouse model with tumor, we employed artificially constructed “cold” pancreatic cancer cells expressing high levels of CLDN18.2 and isolated macrophages from CD40-humanized transgenic mouse. Such design facilitates enhance the clinical relevance of our research.

## Conclusions

In this study, we have successfully developed two scFv antibodies (i.e., anti-CD40 scFv and anti-CLDN18.2 scFv) on the cell membrane through a genetical engineering approach. Using the scFv-anchored cell membrane, we have further engineered nanoengagers with the capacity to enhance the specific recognition and phagocytosis of tumor cells by macrophages. These nanoengagers exhibited remarkable anti-tumor efficacy against pancreatic cancer, concurrently enhancing the infiltration of effector T cells into the tumor microenvironment. Beyond the scope of this study, the adaptable design of bispecific nanoengagers offers the opportunity to advance immunotherapeutic strategies by facilitating crosslinking between tumor cells and other immune cells, such as T cells. This adaptability opens avenues for the exploration of novel and targeted approaches in cancer immunotherapy.

### Electronic supplementary material

Below is the link to the electronic supplementary material.

**Supplementary Material 1**: **Figure S1**. Flow cytometry analysis for determining the expression of CLDN18.2 on the KPC cells and OVA on KPC-CLDN18.2 cells. Flow cytometry results showing the EGFP expression on the KPC cells (a), human claudin 18.2 expression on the KPC cells (b), mCherry expression on the KPC-CLDN18.2-OVA cells (c), and the OVA peptide epitope expression on the KPC-CLDN18.2-OVA cells (d). **Figure S2**. Cognate T cells activated by NanoBE. (a) Illustration of T cells activation after BMMs phagocytosis-mediated by NanoBE. (b) IFN-γ secretion by T cells measured by ELISA kit assay. **Figure S3**. The flow cytometry gating strategies for in vivo experiments. **Figure S4**. Representative H&E staining images of liver. Scale bar=100 μm. **Table S1**. The amino acid sequences of anti-CLDN18.2 scFv and OVA.

## Data Availability

The data and antibody sequences that support the findings of this study are available from the corresponding authors upon reasonable request.
