# Supplementary material for: Genetically engineered membrane-based nanoengagers for immunotherapy of pancreatic cancer
Source: J Nanobiotechnology. 2024 Mar 11;22:104. doi: 10.1186/s12951-024-02369-9 (PMC10926568; doi:10.1186/s12951-024-02369-9)
Supplement: Supplementary file 1 — Supplementary Material 1: Figure S1. Flow cytometry analysis for determining the expression of CLDN18.2 on the KPC cells and OVA on KPC-CLDN18.2 cells. Flow cytometry results showing the EGFP expression on the KPC cells (a), human claudin 18.2 expression on the KPC cells (b), mCherry expression on the KPC-CLDN18.2-OVA cells (c), and the OVA peptide epitope expression on the KPC-CLDN18.2-OVA cells (d). Figure S2. Cognate T cells activated by NanoBE. (a) Illustration of T cells activation after BMMs phagocytosis-mediated by NanoBE. (b) IFN-γ secretion by T cells measured by ELISA kit assay. Figure S3. The flow cytometry gating strategies for in vivo experiments. Figure S4. Representative H&E staining images of liver. Scale bar=100 μm. Table S1. The amino acid sequences of anti-CLDN18.2 scFv and OVA. [file 12951_2024_2369_MOESM1_ESM.docx]

Supporting Information

**Genetically Engineered Membrane-based Nanoengagers for Immunotherapy of Pancreatic Cancer**

Haoqi Zhang^1,†^, Yuanke Li^1,†^, Helong Kang^1^, Jingping Lan^1^, Lin Hou^1^, Zhengbang Chen^2^, Fan Li^1^, Yanqin Liu^1^, Jiliang Zhao^1^, Na Li^1^, Yajuan Wan^1^, Yiping Zhu^1^, Zhen Zhao^3^, Hongkai Zhang^1^, Jie Zhuang^2,*^, Xinglu Huang^1,*^

^1^State Key Laboratory of Medicinal Chemical Biology, Key Laboratory of Bioactive Materials for the Ministry of Education, College of Life Sciences, and Frontiers Science Center for Cell Responses, Nankai University, Tianjin 300071, China.

^2^School of Medicine, Nankai University, Tianjin 300071, China.

^3^Key Laboratory of Molecular Biophysics of Hebei Province, Institute of Biophysics, School of Health Sciences and Biomedical Engineering, Hebei University of Technology, Tianjin 300401, China.

^*^Corresponding authors: [zhuangj@nankai.edu.cn](mailto:zhuangj@nankai.edu.cn); [huangxinglu@nankai.edu.cn](mailto:huangxinglu@nankai.edu.cn);

^†^These authors contributed equally to this work.


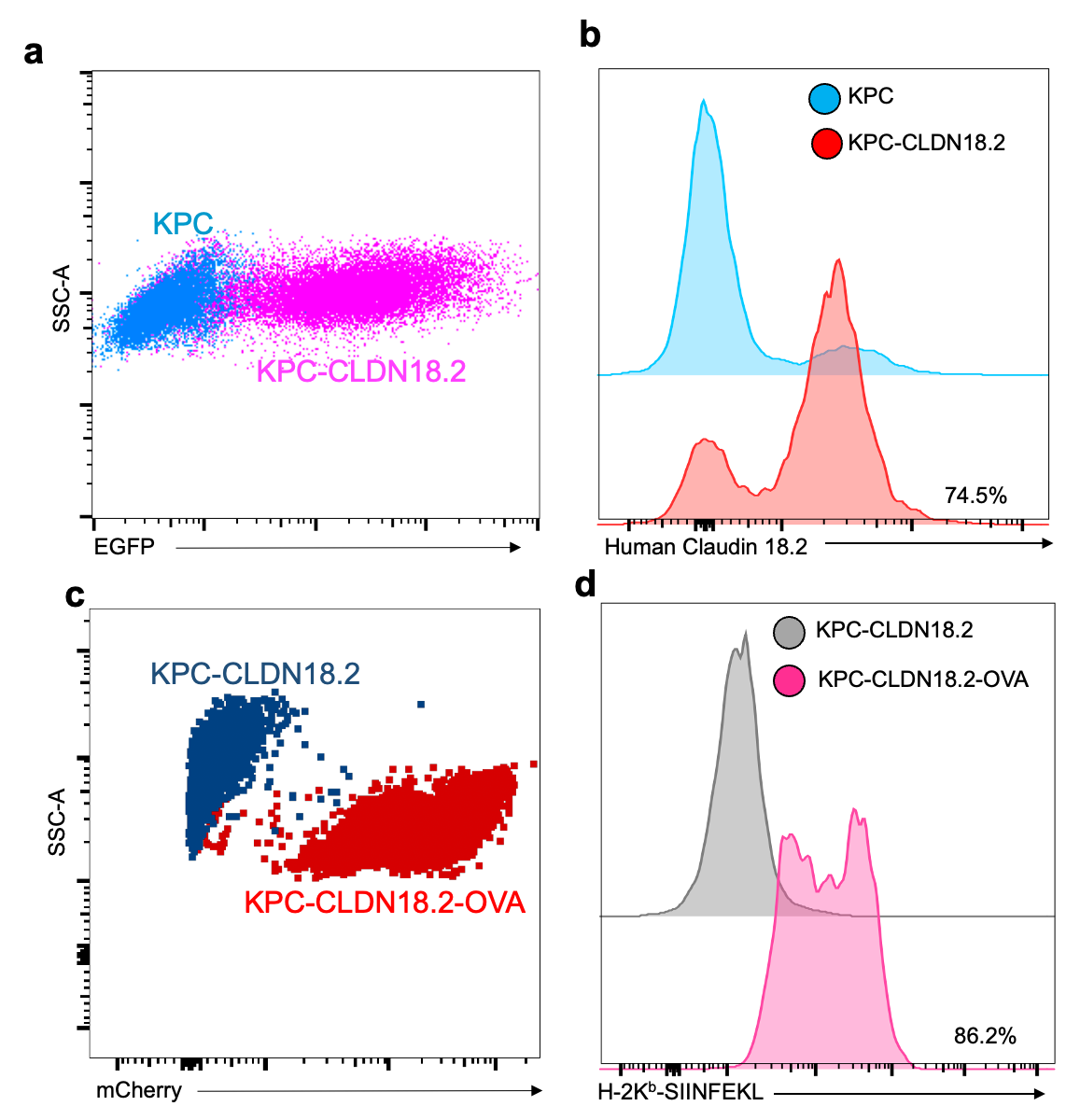


**Figure S1. Flow cytometry analysis for determining the expression of CLDN18.2 on the KPC cells and OVA on KPC-CLDN18.2 cells.** Flow cytometry results showing the EGFP expression on the KPC cells (a), human claudin 18.2 expression on the KPC cells (b), mCherry expression on the KPC-CLDN18.2-OVA cells (c), and the OVA peptide epitope expression on the KPC-CLDN18.2-OVA cells (d).


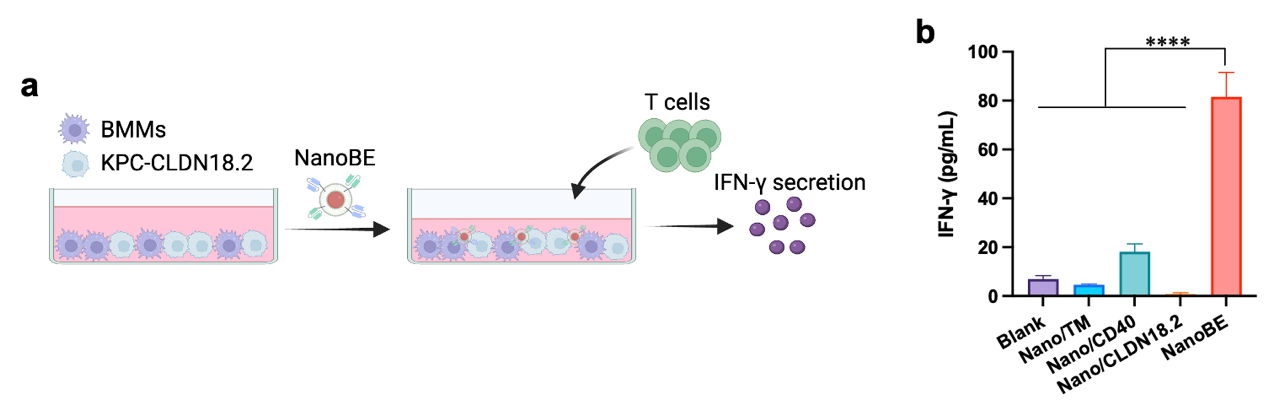
**Figure S2. Cognate T cells activated by NanoBE**. (a) Illustration of T cells activation after BMMs phagocytosis-mediated by NanoBE. (b) IFN-γ secretion by T cells measured by ELISA kit assay.


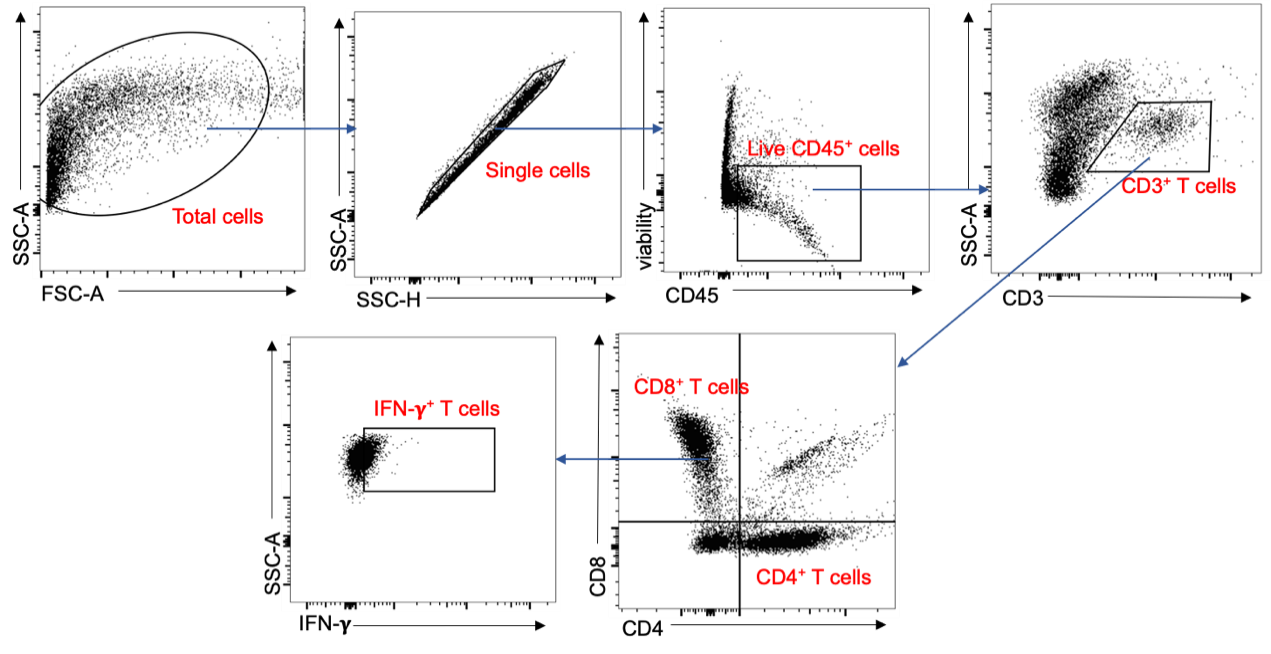


**Figure S3. The flow cytometry gating strategies for *in vivo* experiments.**


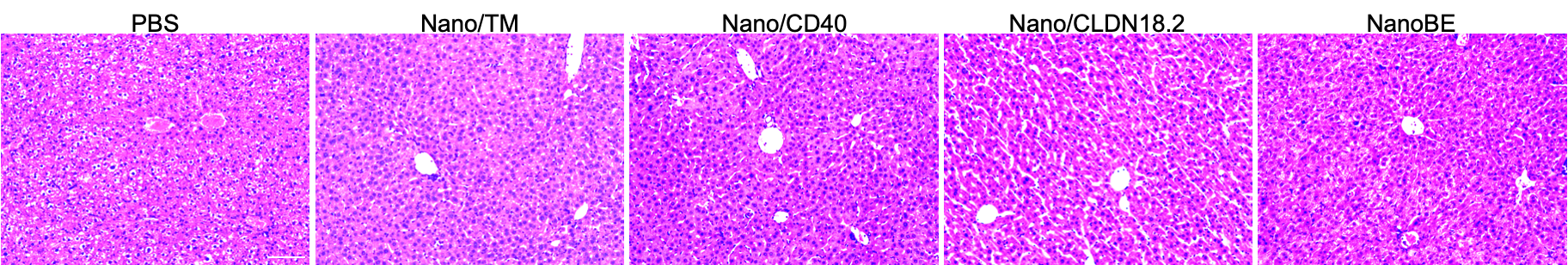


**Figure S4. Representative H&E staining images of liver.** Scale bar=100 µm.

**Table S1. The amino acid sequences of anti-CLDN18.2 scFv and OVA**
